# Adaptation to abiotic conditions drives local adaptation in bacteria and viruses coevolving in heterogeneous environments

**DOI:** 10.1098/rsbl.2015.0879

**Published:** 2016-02

**Authors:** Florien A. Gorter, Pauline D. Scanlan, Angus Buckling

**Affiliations:** 1 Laboratory of Genetics, Department of Plant Sciences, Droevendaalsesteeg 1, 6708PB Wageningen, The Netherlands; 2 Teagasc Food Research Centre, Moorepark, Fermoy, Co. Cork, Ireland; 3 ESI & CEC, Biosciences, University of Exeter, Penryn Campus, Cornwall TR10 9EZ, UK

**Keywords:** host–parasite interactions, local adaptation, coevolution, bacteria, bacteriophage, environmental heterogeneity

## Abstract

Parasite local adaptation, the greater performance of parasites on their local compared with foreign hosts, has important consequences for the maintenance of diversity and epidemiology. While the abiotic environment may significantly affect local adaptation, most studies to date have failed either to incorporate the effects of the abiotic environment, or to separate them from those of the biotic environment. Here, we tease apart biotic and abiotic components of local adaptation using the bacterium *Pseudomonas fluorescens* and its viral parasite bacteriophage Φ2. We coevolved replicate populations of bacteria and phages at three different temperatures, and determined their performance against coevolutionary partners from the same and different temperatures. Crucially, we measured performance at different assay temperatures, which allowed us to disentangle adaptation to biotic and abiotic habitat components. Our results show that bacteria and phages are more resistant and infectious, respectively, at the temperature at which they previously coevolved, confirming that local adaptation to abiotic conditions can play a crucial role in determining parasite infectivity and host resistance. Our work underlines the need to assess host–parasite interactions across multiple relevant abiotic environments, and suggests that microbial adaption to local temperatures can create ecological barriers to dispersal across temperature gradients.

## 1. Introduction

Parasite local adaptation, the greater performance of parasites on their local compared with foreign hosts, has important consequences for the maintenance of diversity and epidemiology [1]. However, in natural environments, local adaptation is likely to be shaped not only by the interaction between host and parasite genotypes, but also by the physical environment [2]. While understanding what determines local adaptation is clearly crucial for understanding its consequences, the two typical approaches to measuring local adaptation necessarily fail to do this. On the one hand, reciprocal transplant studies, in which a genotype's performance is assessed in multiple natural environments, include the effect of the abiotic environment, but do not distinguish between adaptation to biotic and abiotic habitat components. On the other hand, common-garden experiments ignore the abiotic environment altogether. Ideally, host and parasite genotype's as well as the abiotic environment would be manipulated in factorial designs to determine the importance of each of these factors [3]. We are aware of only two empirical studies that attempt to tease apart biotic and abiotic components of local adaptation using plant–pathogen systems, but the scale of these experiments (three host–parasite communities [4] and two host wheat cultivars [5]) necessarily limited the conclusions that could be drawn. Here we tease apart biotic and abiotic components of local adaptation using experimentally coevolving populations of the psychrotrophic soil bacterium *Pseudomonas fluorescens* and its viral parasite lytic bacteriophage SBW25Φ2 [6].

*Pseudomonas fluorescens* and phage readily and continually coevolve under standard laboratory conditions (i.e. in glass vials containing rich liquid medium incubated at 28°C), but it is increasingly clear that results are critically dependent on the abiotic experimental conditions [7,8]. One of the most ubiquitous sources of abiotic variation in nature is temperature. Changes in temperature have profound consequences for the composition of microbial communities [9,10], and given the prominence of climate change, it is particularly important to understand the effect of temperature on (microbial) coevolution. Temperature has major effects on *P. fluorescens* and phage: *P. fluorescens* growth increases with temperature [11], and almost 40% of its genes are thermoregulated [12]. Conversely, temperatures above 28°C significantly reduce phage fitness [13], although it is unclear how phage is affected by lower temperatures.

We coevolved replicate populations of *P. fluorescens* and Φ2 at three different temperatures (8°C, 17°C and 28°C), which closely match the range of yearly average soil temperatures encountered across the USA [14]. We then determined resistance/infectivity of coevolved isolates against coevolutionary partners from the same and different temperatures. Crucially, we measured performance at different assay temperatures, which allowed us to disentangle adaptation to abiotic and biotic habitat components. We anticipated that this design would reveal local adaptation to both the abiotic and the biotic environment. That is, we expected that bacteria and phages would perform better at the temperature at which they coevolved, and that either bacteria or phages would perform better against coevolutionary partners from the same, compared with different, temperatures.

## 2. Material and methods

### (a) Selection experiment

*Pseudomonas fluorescens* SBW25 and bacteriophage SBWΦ2 were cultured in liquid King's medium B (KB) for 10 transfers as previously described [6]. Six replicate coevolving populations were grown at each of three different temperatures: 8°C, 17°C and 28°C. Every 48 h, 1% of each population was transferred to a new tube; optical density (OD_600_) was determined every second transfer as a surrogate measure of bacterial density. As a control, we cultured six replicate bacterial populations at each temperature without phages. After 10 transfers, we isolated 10 bacterial clones per coevolving population by plating out dilutions on KB agar. Phage populations were isolated using chloroform extraction [6].

### (b) Resistance assays

Bacteria were grown up at the temperature at which they coevolved and used to make soft agar plates. Each bacterium was then tested for resistance against its sympatric phage population and an allopatric phage population from the same temperature, as well as one phage population from both other temperatures. Assays were performed at the coevolutionary temperature of the bacterium, as well as the coevolutionary temperature of the phage population (table 1). In each case, 5 µl of phage stock was spotted onto the soft agar plate, and the bacterium was scored as resistant if no plaques were observed after 3 days (8°C) or 1 day (17°C and 28°C).

**Table 1.** Combinations of coevolved bacteria and phages from different temperatures (*T*_B_ and *T*_V_) assayed at each temperature (*T*_A_). Asterisks indicate combinations for which both a sympatric and an allopatric bacterium–phage combination were assayed.

	*T*_A_ (°C)								
	**8**			**17**			***28***		
	*T*_V_ (°C)			T_V_ (°C)			T_V_ (°C)		
*T*_B_ (°C)	8	17	28	8	17	28	8	17	28
8	X*	X	X		X				X
17	X			X	X*	X			X
28	X				X		X	X	X*

### (c) Data analyses

Resistance was calculated as the proportion of bacteria (out of 10) per population that was not infected by phages; conversely, infectivity is the proportion of bacteria that was infected. The log-transformed OD_600_ and arcsine-transformed resistance/infectivity were analysed using linear mixed models.

## 3. Results

**Figure 1.**
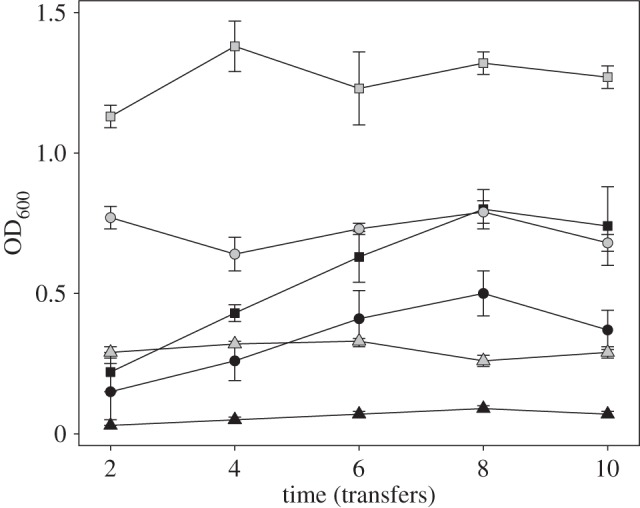
Mean (±s.e.m.) optical density (OD_600_) of evolving (*P. fluorescens*) and coevolving (*P. fluorescens* and SBW25Φ2) populations over time. Populations evolved at different temperatures (triangles: 8°C, circles: 17°C, squares: 28°C) in the absence (grey symbols) or presence (black symbols) of phages.

Bacterial densities (OD_600_) increased with temperature but decreased in the presence of phages (figure 1; effect of temperature: *F*_2,30_ = 109.66, *p* < 0.0001; effect of phages: *F*_1,30_ = 153.05, *p* < 0.0001). Over the course of the selection experiment, bacterial densities increased for coevolving but not evolving populations (phages × time: *F*_1,142_ = 42.86, *p* < 0.0001), and this effect was the same for all temperatures (temperature × phages × time: *F*_2,138_ = 0.80, *p* = 0.4530). This increase in density in the coevolving populations resulted from the evolution of resistance of ancestral SBW25 to phages (see below), against which the ancestral strain of bacteria is sensitive.

**Figure 2.**
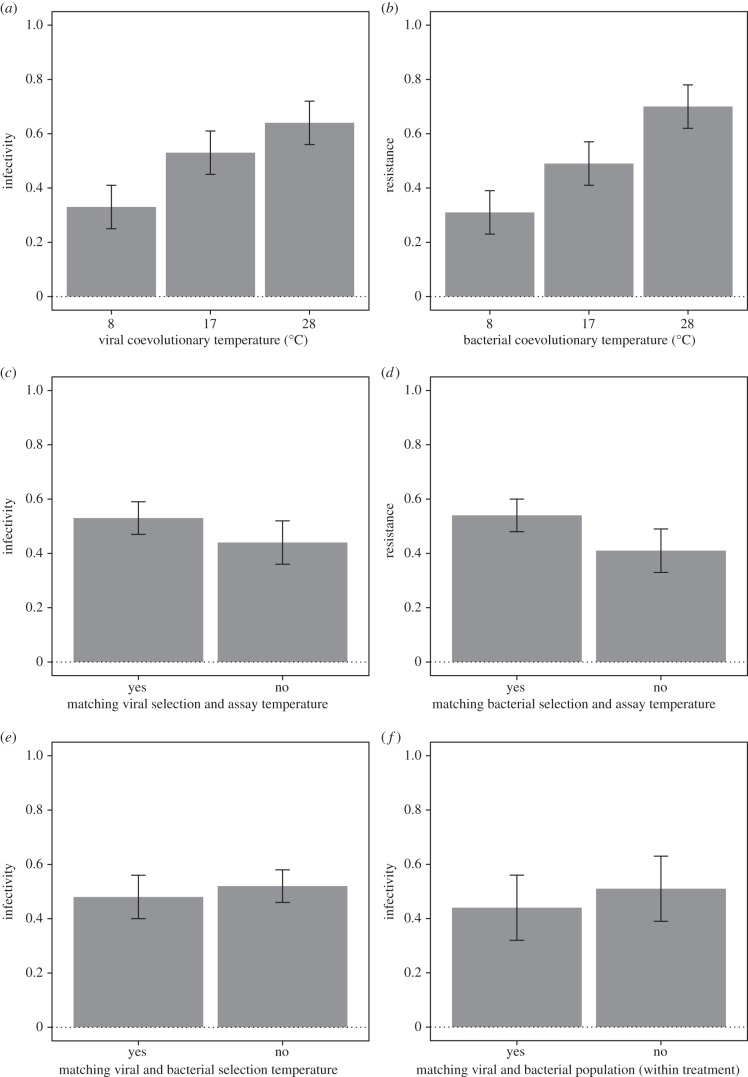
Performance of coevolved phages and bacteria. (*a*) Mean (±s.e.m.) infectivity (i.e. proportion of bacteria that a phage population could infect) of phages coevolved at different temperatures. (*b*) Mean (±s.e.m.) resistance (i.e. proportion of bacteria that could resist viral infection) of bacteria coevolved at different temperatures. (*c*) Mean (±s.e.m.) infectivity of phages when assayed at their selection temperature versus at a different temperature. (*d*) Mean (±s.e.m.) resistance of bacteria when assayed at their selection temperature versus at a different temperature. (*e*) Mean (±s.e.m.) infectivity of phages against bacteria from the same versus a different selection temperature. (*f*) Mean (±s.e.m.) infectivity of phages against bacteria from the same versus a different replicate coevolving population (i.e. infectivity against sympatric versus allopatric bacteria), using only data from within each temperature treatment.

Performance of coevolved populations of phages (bacteria) in terms of infectivity (resistance) against their sympatric bacteria (phages) as well as allopatric bacteria (phages) from all treatments was assessed at both the viral and the bacterial coevolution temperature. Phages that coevolved at higher temperatures were more infectious (figure 2*a* and electronic supplementary material, figure S1*a*–*c*; effect of viral coevolutionary temperature: *F*_1,12_ = 7.17, *p* = 0.0234). Similarly, bacteria from higher temperatures were more resistant (figure 2*b* and electronic supplementary material, figure S1*d*–*f*; effect of bacterial coevolutionary temperature: *F*_1,14_ = 14.25, *p* = 0.0106). Conversely, there was no main effect of assay temperature on performance (*F*_2,86_ = 0.25, *p* = 0.7761).

Both phages and bacteria became locally adapted to their abiotic environment, that is, they performed significantly better at the temperature at which they coevolved (phages: *F*_1,71_ = 4.63, *p* = 0.0349, figure 2*c* and electronic supplementary material, figure S1*a*–*c*; bacteria: *F*_1,71_ = 5.23, *p* = 0.0215, figure 2*d* and electronic supplementary material, figure S1*d*–*f*). However, neither bacteria nor phages were consistently locally adapted (or maladapted) to coevolutionary partners from the same versus different coevolutionary temperature (*F*_1,71_ = 0.02, *p* = 0.8805; figure 2*e*) or to populations they actually coevolved with (effect of sympatry: *F*_1,73_ = 0.70, *p* *=* 0.4069; figure 2*f*).

## 4. Discussion

Our results show that *P. fluorescens* and bacteriophage Φ2 are significantly more resistant and infectious, respectively, at the temperature at which they coevolved. That is, both bacteria and phages were locally adapted to their abiotic environment. Conversely, we found no indication for local adaptation to biotic habitat components: neither bacteria nor phages performed better (or worse) against phages and bacteria evolved at the same versus different temperatures, or against their sympatric, versus allopatric, coevolutionary partners. These results show that adaptation to temperature *per se* is likely to play a more important role in determining phage local adaptation than adaptation to hosts that coevolved at the same temperature. More generally, these results, along with the finding that bacteria and phages that coevolved at higher temperatures were more resistant and infectious, respectively, show that adaptation to abiotic conditions can play a crucial role in determining parasite infectivity and host resistance. This is broadly consistent with previous work on a plant–pathogen system, where there was evidence that fungal pathogens are adapted to their selection temperatures [4,5].

Unlike this study, a previous study from this system found that coevolved phages were locally adapted to bacteria from the same abiotic conditions [8]. However, in that case abiotic variation was created by nutrient concentration. Part of the explanation for this discrepancy may be that local adaptation to biotic habitat components was present in our study, but that it was obscured by the large differences in overall performance between coevolved isolates from different temperatures. Indeed, in the previous study, where a wide range of nutrient conditions was used, local adaptation was obscured between environments where there were large differences in mean infectivity/resistance. It is also likely that different abiotic environments affect the relative importance of biotic versus abiotic adaptation. For example, the expression of surface receptors on bacterial cells to which phages adsorb may be more strongly affected by nutrient availability than temperature, hence increasing the importance of biotic adaptation in the former.

Coevolutionary temperature had a clear effect on the final level of resistance and infectivity of coevolved bacteria and phages, with the highest levels corresponding to the highest temperatures. This may be explained by the smaller bacterial population sizes at lower temperatures, which are likely to have limited mutation supply rate and thus the rate of coevolution [15]. It is also possible that the different rates of resistance/infectivity resulting from different coevolutionary temperatures reflect not different rates of coevolution, but different types of coevolutionary dynamic (arms race dynamics versus fluctuating selection) [7], which environmental conditions are known to affect.

Our work confirms the need to assess host–parasite interactions across multiple environments in which populations coevolve. Studies that assess performance in a single environment may considerably underestimate local adaptation: in our case, we would not have found any consistent patterns of local adaptation if we had measured performance in a single environment. Indeed, common-garden designs are more likely to find local maladaptation than reciprocal transplant designs [16]. Equally problematic are reciprocal transplant experiments, which cannot determine the relative importance of the factors driving local adaptation [3]. This is crucial, because the ecological and evolutionary consequences of local adaptation will be unknown. For example, the consequences for hosts moving into a new environment will be fundamentally different depending on whether the local, or resident, parasites in this new environment are primarily adapted to the local host genotypes or to the local physical environment. Our work has more specific implications for microbial and viral ecology, suggesting that the propensity for viruses (and microbes) to adapt to local temperatures can create ecological barriers to dispersal across temperature gradients.

## Supplementary Material

Supplementary material

## Data accessibility

Data are available at the Dryad Digital Repository: http://dx.doi.org/10.5061/dryad.7j1nr.
