# Supplementary material for: Adaptation to abiotic conditions drives local adaptation in bacteria and viruses coevolving in heterogeneous environments
Source: Biol Lett. 2016 Feb;12(2):20150879. doi: 10.1098/rsbl.2015.0879 (PMC4780547; doi:10.1098/rsbl.2015.0879)
Supplement: Supplementary material [file rsbl20150879supp1.docx]

**Supplementary material for Gorter et al. - Biology Letters**

**Data analyses**

To identify the factors affecting bacterial population size, we fitted general linear mixed models (GLMs) with log-transformed optical density (OD_600­_) as the response variable. Transformation was employed to correct for increasing variance, and because we were interested in relative rather than absolute changes in population size. (Co)evolutionary temperature (three levels: 28°C, 17°C, and 8°C), the presence of phages (two levels: yes/ no), time in transfers, and all interactions between these factors were used as fixed effects, while replicate (co)evolution line was used as a random effect. We used sequential backwards elimination to find the minimal adequate model, and report relevant statistics from the minimal model containing each effect.

GLMs were also used to determine whether resistance, that is, the arcsine transformed proportion of resistant bacteria per population, was affected by bacterial coevolutionary temperature, viral coevolutionary temperature, assay temperature (three levels: 28°C, 17°C, and 8°C), and sympatry (two levels: yes/ no). Both bacterial and viral replicate coevolution line were used as random effects. Statistics are reported as above. The effect of each interaction term (that is, “bacterial coevolutionary temperature = assay temperature”, “viral coevolutionary temperature = assay temperature”, and “bacterial coevolutionary temperature = viral coevolutionary temperature”, each with two levels: yes/ no) was separately assessed using models containing all main effects and the interaction term.

All statistical analyses were performed in R [26].

**Figure S1.** Performance of coevolved phages and bacteria. Mean (± SEM) infectivity (i.e. proportion of bacteria that a phage population could infect) of phages coevolved at 8°C (A), 17°C (B), and 28°C (C) versus bacteria coevolved at different temperatures, assayed at the viral (dark bars) or bacterial (light bars) coevolutionary temperature. Mean (± SEM) resistance (i.e. proportion of bacteria that could resist viral infection) of bacteria coevolved at 8°C (D), 17°C (E), and 28°C (F) versus phages coevolved at different temperatures, assayed at the bacterial (dark bars) or viral (light bars) coevolutionary temperature. In other words, dark bars show infectivity or resistance where assay and abiotic selection environment match.
